# Supplementary material for: Biallelic DAW1 variants reveal a tissue-specific role in heterotaxy without primary ciliary dyskinesia
Source: Eur J Hum Genet. 2026 Sep 22;34(11):1693–705. doi: 10.1038/s41431-026-02228-1 (PMC13743782; doi:10.1038/s41431-026-02228-1)
Supplement: Supplementary file 1 [file 41431_2026_2228_MOESM2_ESM.docx]

**SUPPLEMENTAL FIGURE LEGEND**

**Figure S1. SpliceAI prediction of DAW1 c.648+1G>A consequences.**

SpliceAI Δ scores for the c.648+1G>A variant computed against transcript ENST00000309931.3 (NM_178821.3). Δ Donor Loss = 0.95 at −1 bp indicates near-complete disruption of the natural splice donor at the exon 7 / intron 7 boundary. Δ Acceptor Loss = 0.54 at −108 bp indicates concomitant weakening of the exon 7 acceptor signal. Δ Acceptor Gain = 0.00 and Δ Donor Gain = 0.06 (at +459 bp) are both well below the standard 0.5 SpliceAI threshold, providing strong evidence against activation of cryptic splice sites. Together, these scores support exon 7 skipping as the dominant predicted splicing outcome, resulting in the in-frame deletion p. Val181_Arg216del, which is predicted to escape nonsense-mediated decay.

**SUPPLEMENTAL TABLE**

**Table S1: Summary of all reported DAW1 variants and their associated phenotypes.**
Previously reported families in Leslie et al., 2022 and Jin et al., 2017 include individuals with homozygous and compound heterozygous variants, presenting with variable features of laterality defects, congenital heart disease (CHD), and respiratory issues. Functional assays in zebrafish distinguished complete loss-of-function alleles (e.g., p.(Asn143Asp), p.(Ser364Thr)) from hypomorphs (e.g., p.(Trp372Cys)). The proband in this study carried compound heterozygous variants (p.(Arg114Gln)/splice-site c.648+1G>A) and exhibited HTX/laterality defect spectrum, presenting as complex CHD, without respiratory disease. *Xenopus tropicalis* assays showed that p.(Arg114Gln) functions as a context-specific hypomorph affecting LRO cilia but not MCCs, while the splice-site variant indicates a complete loss-of-function. These results collectively expand the DAW1-related disease spectrum.

| **Family** | **Zygosity** | **cDNA Change** | **Protein Change** | **Phenotype** | **Functional Evidence** | **Reference** |
| --- | --- | --- | --- | --- | --- | --- |
| **1** | Hom | c.427A>G | p.(Asn143Asp) | Situs inversus (2 patients, no respiratory); 1 patient respiratory only | Zebrafish LOF (no rescue of cardiac looping or KV motility) | Leslie et al., 2022 |
| **2** | Hom | c.356G>A | p.(Trp119*) | Complex CHD, situs ambiguous, transverse liver, right-sided spleen; no respiratory | Predicted LOF nonsense allele | Leslie et al., 2022 |
| **3** | Hom | c.1091G>C | p.(Ser364Thr) | Complex CHD resembling HTX; respiratory unknown | Zebrafish LOF (no rescue of cardiac looping or KV motility) | Leslie et al., 2022, Jin et al., 2017 |
| **4** | Cmp Het | c.197T>A/c.1116G>T | p.(Leu66*)/p.(Trp372Cys) | Complex CHD resembling HTX; respiratory unknown | Zebrafish partial hypomorph (rescue incomplete) with Trp372Cys | Leslie et al., 2022, Jin et al., 2017 |
| **This study** | Cmp Het | c.341G>A / c.648+1G>A | p.(Arg114Gln) / splice | HTX/laterality defect spectrum, presenting as complex CHD; no PCD/respiratory disease | *Xenopus*: context-specific hypomorph (R114Q LRO defect, MCC intact); splice LOF | This study |

# **Table S2. Prioritized candidate genes from whole-genome sequencing analysis.**

Variants identified from whole-genome sequencing were prioritized using Genomiser with the following Human Phenotype Ontology (HPO) terms: *Double outlet right ventricle* (HP:0001719), *Dextrotransposition of the great arteries* (HP:0031348), and *Subarterial ventricular septal defect* (HP:0011681). All gene–mode-of-inheritance combinations with non-zero phenotype similarity scores are listed. *DAW1* ranked first under autosomal recessive inheritance and was selected as the top candidate. All other candidates were excluded based on incompatible inheritance, lack of qualifying variants that meet pathogenicity filters, or substantial phenotype mismatch, as detailed in the rightmost column. MOI: mode of inheritance; AR: autosomal recessive; AD: autosomal dominant; XR: X-linked recessive; XD: X-linked dominant.

| **Rank** | **Gene** | **MOI** | **OMIM Phenotype** | **Combined Score** | **Pheno Score** | **Reason for Exclusion / Selection** |
| --- | --- | --- | --- | --- | --- | --- |
| **1** | ***DAW1*** | AR | Ciliary dyskinesia, primary, 52 (OMIM:620570) | 0.876 | 0.683 | **Selected as top candidate. Compound heterozygous variants segregating with autosomal recessive inheritance; HPO matches include double-outlet right ventricle, transposition of the great arteries, and ventricular septal defect.** |
| 2 | *RYR1* | AD | King–Denborough syndrome (OMIM:619542) | 0.783 | 0.522 | Excluded. Dominant inheritance is incompatible with two unaffected parents lacking myopathy or malignant hyperthermia susceptibility. Phenotype mismatch: RYR1-AD primarily causes skeletal myopathy with malignant hyperthermia susceptibility, not laterality defects. HPO overlap limited to non-specific ventricular septal defect. |
| 3 | *KAT8* | AD | Li–Ghorgani–Weisz–Hubshman syndrome (OMIM:618974) | 0.415 | 0.546 | Excluded. Dominant inheritance incompatible with healthy parents. Phenotype mismatch: characterized by intellectual disability, dysmorphic features, and seizures, none of which are present in the proband. HPO overlap limited to non-specific patent ductus arteriosus. |
| 4 | *CCDC22* | XR | Ritscher–Schinzel syndrome 2 (OMIM:300963) | 0.413 | 0.538 | Excluded. Although X-linked recessive inheritance is consistent with hemizygosity in a male, the phenotype is dominated by craniofacial anomalies, cerebellar hypoplasia, and intellectual disability — none observed in the proband. HPO overlap is limited to non-specific cardiac features. |
| 5 | *RYR1* | AR | King–Denborough syndrome (OMIM:619542) | 0.353 | 0.522 | Excluded. Same phenotype mismatch as RYR1-AD. No qualifying biallelic variants meeting filtering thresholds were identified to support a recessive inheritance call. |
| 6 | *DAW1* | AD | Ciliary dyskinesia, primary, 52 (OMIM:620570) | 0.016 | 0.683 | Same gene as Rank 1 but evaluated under autosomal dominant inheritance; superseded by the higher-confidence AR call (Rank 1). |
| 7 | *ALG12* | AD | ALG12-CDG (ORPHA:79324) | 0.015 | 0.534 | Excluded. Dominant inheritance incompatible with healthy parents. Congenital disorder of glycosylation phenotype includes intellectual disability, hypogonadism, and immunodeficiency — none observed in the proband. |
| 8 | *DNAH1* | AR | Primary ciliary dyskinesia (ORPHA:244) | 0.003 | 0.642 | Excluded. While DNAH1 is a known motile ciliopathy gene with relevant phenotype overlap, the variant score was very low (0.0028); no qualifying biallelic variants meeting pathogenicity filters were identified. |
| 9 | *CCDC22* | XD | Ritscher–Schinzel syndrome 2 (OMIM:300963) | 0.003 | 0.538 | Excluded. X-linked dominant inheritance unlikely given unaffected mother; phenotype mismatch as described above. |
| 10 | *PSMD12* | AD | Stankiewicz–Isidor syndrome (OMIM:617516) | 0.002 | 0.573 | Excluded. Dominant inheritance incompatible with healthy parents. Phenotype includes intellectual disability and dysmorphism, absent in proband. |
| 11 | *ARID1B* | AD | Coffin–Siris syndrome (ORPHA:1465) | 0.001 | 0.539 | Excluded. Dominant inheritance incompatible with healthy parents. Phenotype dominated by intellectual disability, coarse facies, and fifth-digit anomalies — none present in proband. |
| 12 | *PALB2* | AD | Fanconi anemia, complementation group N (OMIM:610832) | 0.0001 | 0.527 | Excluded. Phenotype mismatch: Fanconi anemia is a hematologic and developmental disorder with growth failure, bone marrow failure, and cancer predisposition. |
| 13 | *PRDM13* | AD | Pontocerebellar hypoplasia, type 17 (OMIM:619909) | 0.000 | 0.546 | Excluded. Combined score effectively zero. Phenotype mismatch: cerebellar/brainstem developmental disorder absent in proband. |
| 13 | *CHMP1A* | AD | Pontocerebellar hypoplasia, type 8 (OMIM:614961) | 0.000 | 0.525 | Excluded. Combined score effectively zero. Phenotype mismatch as for PRDM13. |
| 13 | *ARID1B* | AR | Coffin–Siris syndrome (ORPHA:1465) | 0.000 | 0.539 | Excluded. Same phenotype mismatch as ARID1B-AD; no qualifying biallelic variants identified. |
| 13 | *SON* | AR | ZTTK syndrome (OMIM:617140) | 0.000 | 0.524 | Excluded. ZTTK syndrome is a dominant disorder with intellectual disability and dysmorphism; AR call here reflects ranking artifact rather than biological plausibility. |
| 13 | *FBN2* | AR | Contractural arachnodactyly, congenital (OMIM:121050) | 0.000 | 0.532 | Excluded. Connective tissue disorder phenotype (arachnodactyly, contractures, kyphoscoliosis) absent in proband. |

Combined and phenotype scores are direct outputs from Genomiser/Exomiser. Variants were filtered using recommended best-practice settings (REVEL, MVP, AlphaMissense, and SpliceAI as variant pathogenicity prediction sources; human-only hiPHIVE gene–phenotype associations; ClinVar whitelist; inheritance filters) as described in Methods.
